# Relationship between molecular response and quality of life with bosutinib or imatinib for chronic myeloid leukemia

**DOI:** 10.1007/s00277-020-04018-1

**Published:** 2020-04-19

**Authors:** Tim H. Brümmendorf, Carlo Gambacorti-Passerini, Andrew G. Bushmakin, Joseph C. Cappelleri, Andrea Viqueira, Arlene Reisman, Susanne Isfort, Carla Mamolo

**Affiliations:** 1 Universitätsklinikum RWTH Aachen, Pauwelsstrasse 30, 52074 Aachen, Germany; 2 University of Milano-Bicocca, Monza, Italy; 3 Pfizer Inc, Groton, CT USA; 4 Pfizer SLU, Madrid, Spain; 5 Pfizer Inc, New York, NY USA

**Keywords:** Chronic myeloid leukemia, Health-related quality of life, Molecular response, Bosutinib, Imatinib

## Abstract

Patients with newly diagnosed chronic phase chronic myeloid leukemia (CP CML) can be effectively treated with tyrosine kinase inhibitors (TKIs) and achieve a lifespan similar to the general population. The success of TKIs, however, requires long-term and sometimes lifelong treatment; thus, patient-assessed health-related quality of life (HRQoL) has become an increasingly important parameter for treatment selection. Bosutinib is a TKI approved for CP CML in newly diagnosed adults and in those resistant or intolerant to prior therapy. In the Bosutinib Trial in First-Line Chronic Myelogenous Leukemia Treatment (BFORE), bosutinib demonstrated a significantly higher major molecular response rate compared with imatinib, with maintenance of HRQoL (measured by the Functional Assessment of Cancer Therapy-Leukemia (FACT-Leu) questionnaire), after 12 months of first-line treatment. We examined relationships between molecular response (MR) and HRQoL. MR values were represented by a log-reduction scale (MRLR; a continuous variable). A repeated-measures longitudinal model was used to estimate the relationships between MRLR as a predictor and each FACT-Leu domain as an outcome. Effect sizes were calculated to determine strength of effects and allow comparisons across domains. The majority of FACT-Leu domains (with the exception of social well-being and physical well-being) demonstrated a significant relationship with MRLR (*p* < 0.05). Our results showed variable impact of clinical improvement on different dimensions of HRQoL. For patients who achieved MR^5^, emotional well-being and leukemia-specific domains showed the greatest improvement, with medium differences in effect sizes, whereas social well-being and physical well-being had the weakest relationship with MR.

## Introduction

Tyrosine kinase inhibitors (TKIs) that block the activity of the *BCR-ABL1* gene fusion product, the hallmark of chronic myeloid leukemia (CML), have substantially improved efficacy and tolerability of treatment and extended patient life expectancy to nearly that of the general population [1]. In clinical trials that compared second-generation TKIs (bosutinib, dasatinib, or nilotinib) with the first-generation TKI imatinib in newly diagnosed patients with chronic phase (CP) CML, the second-generation TKIs showed superior efficacy, defined as cytogenetic or molecular responses (MR) at or by 12 months of treatment, versus imatinib [2–4]. Despite achievement of treatment-free remission becoming an increasingly well-established goal of first-line treatment [5, 6], the majority of patients with CP CML still require lifelong TKI therapy; thus, preserving or improving health-related quality of life (HRQoL) has become an important consideration for treatment selection.

To date, prospective assessment of patient-reported HRQoL in CML trials of most first- and second-generation TKIs has been scarce [7]. However, patient-reported outcome (PRO) data from clinical studies of bosutinib have indicated that patients with CML experienced stable or, in some cases, improved HRQoL during treatment compared with pretreatment status [8–11]. Relationships between distinct side effects of individual TKIs and HRQoL have been explored, e.g., PROs in patients with CML who experienced diarrhea during treatment with bosutinib [10, 12], but associations between efficacy and HRQoL are largely unknown.

Approval of bosutinib for newly diagnosed patients with CP CML was based on data from the ongoing, randomized, phase 3 BFORE trial, which demonstrated a significantly higher major MR (MMR) rate at 12 months in the modified intent-to-treat (ITT) population (primary endpoint) with bosutinib (*n* = 246) versus imatinib (*n* = 241) [2]. After longer follow-up (≥ 24 months), bosutinib continued to demonstrate improved efficacy compared with imatinib, as evidenced by a higher cumulative MMR rate (68.7% vs. 59.3%; odds ratio, 1.51; 95% confidence interval, 1.06–2.16) and MR^4^ rate (39.9% vs. 31.3%; odds ratio, 1.45; 95% confidence interval, 1.02–2.07) in the ITT population (bosutinib, *n* = 268 and imatinib, *n* = 268) at any time on treatment (Pfizer Inc, data on file) [13]. Treatment-emergent adverse events after ≥ 24 months’ follow-up were consistent with the known safety profiles of bosutinib [14] and imatinib [15]; diarrhea and transaminase increases were more frequent with bosutinib, and musculoskeletal events were more common with imatinib (Pfizer Inc., data on file). Assessment of PROs with the Functional Assessment of Cancer Therapy-Leukemia (FACT-Leu) questionnaire was an exploratory objective of the BFORE trial; after 12 months of treatment with either bosutinib or imatinib, patients in the modified ITT population maintained or improved HRQoL compared with baseline [11]. Repeated-measures mixed-effects modeling showed no significant differences in HRQoL at month 12 with bosutinib versus imatinib [11].

Here, we examined the relationships between MR and HRQoL in newly diagnosed patients with CP CML receiving TKI treatment in the BFORE trial, using a pooled analysis of the bosutinib and imatinib arms. To our knowledge, this is the first detailed investigation of the association between efficacy outcomes and HRQoL in this patient population using data from a randomized phase 3 clinical trial.

## Methods

### BFORE study design

As previously described [2], patients eligible for the BFORE study were aged ≥ 18 years and had previously untreated CP CML, with a molecular diagnosis within the previous 6 months, and an Eastern Cooperative Oncology Group performance status of 0 or 1. Patients were randomized 1:1 to receive either bosutinib 400 mg once daily or imatinib 400 mg once daily. Treatment is continued for 5 years until the end of the study or until treatment failure, unacceptable toxicity, death, or withdrawal of consent. The study was conducted in accordance with the Declaration of Helsinki, the protocol was approved by the Institutional Review Board at each study center, and all patients provided written informed consent. The trial is registered on ClinicalTrials.gov (NCT02130557).

### MR and HRQoL assessments

MR was centrally assessed (MolecularMD, Portland, OR) by reverse transcription polymerase chain reaction (RT-PCR) using peripheral blood collected at baseline, every 3 months for the first 24 months of treatment, and every 6 months thereafter, and was evaluated on the international scale [2, 16].

HRQoL was assessed using the patient-reported FACT-Leu (version 4) questionnaire comprising 4 general HRQoL domains (physical, social, emotional, and functional well-being) and a leukemia-specific domain (Table 1) [2, 8, 9, 17, 18]. Each item in the questionnaire was scored from 0 to 4, with higher scores indicating better HRQoL. Three second-order aggregated domains were based on the first-order general HRQoL and leukemia-specific domains: the FACT-General (FACT-G) total score is the sum of the physical, social, emotional, and functional well-being domain scores; the FACT-Leu total score is the sum of the FACT-G total and leukemia-specific domain scores; and the trial outcome index (TOI)-FACT-Leu score is the sum of the physical well-being, functional well-being, and leukemia-specific domain scores, and represents a convenient summary index for clinical trials by assessing outcomes that are likely to change rapidly in response to treatment [18] (Table 1). The minimal important difference (MID), a change that is clinically meaningful to a patient, has been defined as 2–3 points for physical well-being, 2 points for emotional well-being, 2–3 points for functional well-being, 4–7 points for leukemia-specific, 3–7 points for FACT-G, 6–12 points for the FACT-Leu total, and 5–6 points for TOI-FACT-Leu scores [8]; the MID for social well-being has not been defined. Patients were asked to complete FACT-Leu questionnaires at baseline, every 3 months for the first 24 months of treatment, every 6 months thereafter, and at treatment completion [11].

**Table 1:** FACT-Leu questionnaire [18]

Domain	Items, *n*	Score, range	Questions
Physical well-being	7	0–28	• I have a lack of energy • I have nausea • Because of my physical condition, I have trouble meeting the needs of my family • I have pain • I am bothered by side effects of treatment • I feel ill • I am forced to spend time in bed
Social well-being	7	0–28	• I feel close to my friends • I get emotional support from my family • I get support from my friends • My family has accepted my illness • I am satisfied with family communication about my illness • I feel close to my partner (or the person who is my main support) • I am satisfied with my sex life
Emotional well-being	6	0–24	• I feel sad • I am satisfied with how I am coping with my illness • I am losing hope in the fight against my illness • I feel nervous • I worry about dying • I worry that my condition will get worse
Functional well-being	7	0–28	• I am able to work (include work at home) • My work (include work at home) is fulfilling • I am able to enjoy life • I have accepted my illness • I am sleeping well • I am enjoying the things I usually do for fun • I am content with the quality of my life right now
Leukemia-specific	17	0–68	• I am bothered by fevers • I have certain parts of my body where I experience significant pain • I am bothered by the chills • I have night sweats • I am bothered by lumps or swelling in certain parts of my body (e.g., neck, armpits, or groin) • I bleed easily • I bruise easily • I feel weak all over • I get tired easily • I am losing weight • I have a good appetite • I am able to do my usual activities • I worry about getting infections • I feel uncertain about my future health • I worry that I might get new symptoms of my illness • I have emotional ups and downs • I feel isolated from others because of my illness or treatment
Aggregated domain	Items, *n*	Score, range	Calculation
FACT-G total	27	0–108	Sum of the physical well-being, social well-being, emotional well-being, and functional well-being scores
FACT-Leu total	44	0–176	Sum of the FACT-G total and the leukemia-specific scores
TOI-FACT-Leu	31	0–124	Sum of the physical well-being, functional well-being, and the leukemia-specific scores

*FACT-G* Functional Assessment of Cancer Therapy-General, *FACT-Leu* Functional Assessment of Cancer Therapy-Leukemia, *TOI* trial outcome index

### Statistical analysis

Data from the ITT population of patients with newly diagnosed CP CML in both arms of the BFORE trial (bosutinib and imatinib; *n* = 536) were used to examine relationships between MR and patient-reported HRQoL (as measured by FACT-Leu). MR values were represented by a log-reduction scale [19] as a continuous variable.

A repeated-measures longitudinal model [20, 21] was used to estimate the overall relationships between MR log-reduction (MRLR) score (from screening to 24 months) as a predictor and each FACT-Leu domain score as an outcome.

This model incorporated all available data, generally using a heterogeneous autoregressive covariance structure to account for the correlated measurements (i.e., error terms) over time coming from the same individual, where the mechanism of missing data is at random [20, 21]. Even if a patient had a missing observation at a particular post-baseline assessment, the completed post-baseline scores were still considered. To study the appropriateness of the linear approximation of the relationship between predictor and outcome, the model was also evaluated with MRLR score (from screening to 24 months) as a categorical variable, in which MRLR values were rounded to the nearest 0.5 points.

An (standardized) effect size of 0.2 was considered small (i.e., the difference in means of 0.2 baseline standard deviation units), 0.5 was medium, and 0.8 was large; a value of ~ 0.1 was considered trivial [22, 23]. Midpoints between values of 0.1, 0.2, 0.5, and 0.8 were used to create categorization intervals for effect size. In a systematic review of PRO studies, an effect size of ~ 0.5 was determined to be the threshold for detecting changes in HRQoL [24].

## Results

### Relationships between MR and HRQoL in the BFORE trial population

**Fig. 1:**
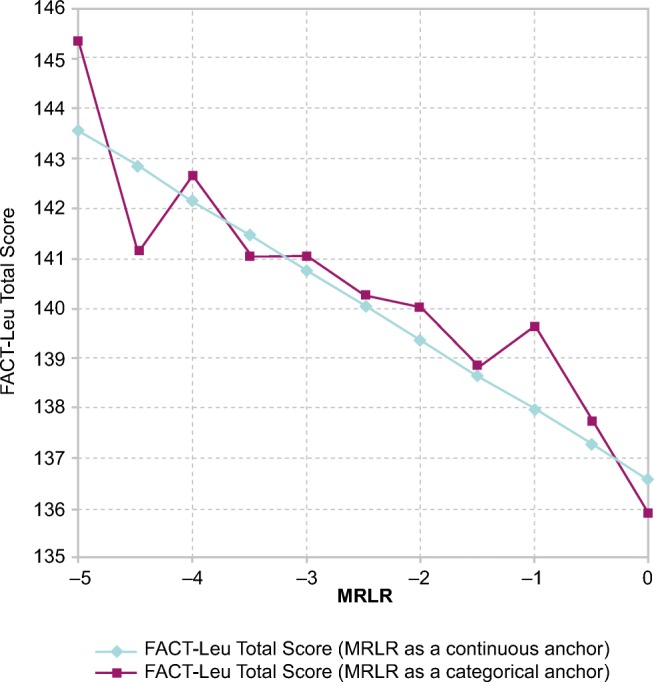
Relationship between MRLR score and FACT-Leu total score in the BFORE trial population. *FACT-Leu* Functional Assessment of Cancer Therapy-Leukemia, *MRLR* molecular response log-reduction

Evaluation of the MRLR score as a continuous versus categorical variable indicated that the linearity assumption for the relationship between MRLR score and FACT-Leu total score was appropriate (Fig. 1). There was some visible departure from linearity for the MRLR value of − 5 and to some extent for the value of − 4.5. This related to the relatively small number of available observations in the model with MRLR score as a categorical predictor, e.g., only 9 (0.34%) observations with an MRLR score of − 5 of 2668 total observations available and used in the analysis. Relationships between MRLR score and other FACT-Leu domain and aggregated domain scores exhibited similar patterns (data not shown).

### Differences in HRQoL according to MR level and interpretation of differences (effect size)

**Fig. 2:**
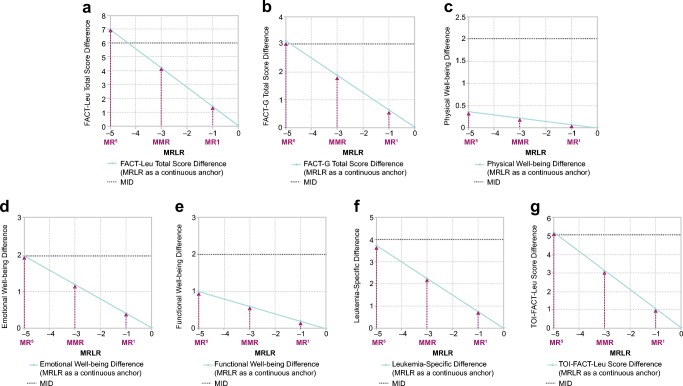
Differences in scores according to MR level for (**a**) FACT-Leu total, (**b**) FACT-G total, (**c**) physical well-being, (**d**) emotional well-being, (**e**) functional well-being, (**f**) leukemia-specific, and (**g**) TOI-FACT-Leu domains. *FACT-G* Functional Assessment of Cancer Therapy-General, *FACT-Leu* Functional Assessment of Cancer Therapy-Leukemia, *MID* minimal important difference, *MMR* major molecular response, *MR* molecular response, *MRLR* molecular response log-reduction, *TOI* trial outcome index

The differences in estimated mean FACT-Leu domain and aggregated domain scores corresponding to MRLR values of − 5 (MR^5^), − 3 (MMR), and − 1 (MR^1^) versus MRLR value of 0 (standardized baseline; no response) in the context of their respective MIDs are shown in Fig. 2; social well-being, for which the MID has not been defined, is not shown. Based on the linear model, FACT-Leu total score differences corresponding to MR^5^, MMR, and MR^1^ were significant (*p* < 0.0001). Differences corresponding to MR^5^, MMR, and MR^1^ were significant (*p* < 0.05) for FACT-G total, emotional well-being, functional well-being, leukemia-specific, and TOI-FACT-Leu scores, but were not significant for physical or social well-being scores. Only patients who achieved a deep MR (MR^5^) exceeded the MID for the FACT-Leu total, FACT-G total, and TOI-FACT-Leu scores. The MID was not reached for physical well-being, emotional well-being, functional well-being, or leukemia-specific scores, regardless of depth of response.

**Fig. 3:**
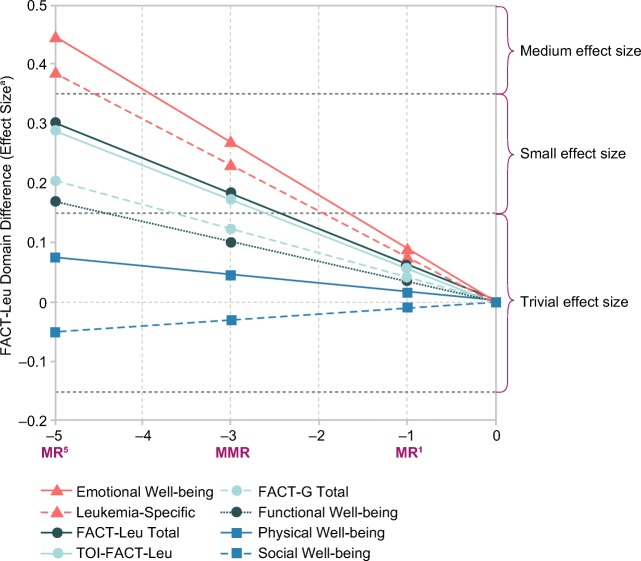
Comparison of the relationships between MR and FACT-Leu domain and aggregated domain scores according to MR level (effect size^a^). *FACT-G* Functional Assessment of Cancer Therapy-General, *FACT-Leu* Functional Assessment of Cancer Therapy-Leukemia, *MMR* major molecular response, *MR* molecular response, *TOI* trial outcome index.^a^ A (standardized) effect size of 0.2 is considered small (i.e., the difference in means being 0.2 baseline standard deviation units), 0.5 medium, and 0.8 large; a value of ~ 0.1 is trivial; midpoints between values of 0.1, 0.2, 0.5, and 0.8 were used to create categorization intervals for effect size

Interpretation of differences, based on effect size, in FACT-Leu domain and aggregated domain scores according to MR level is shown in Fig. 3. MR had the most robust relationships with emotional well-being and leukemia-specific scores, showing medium, small, and trivial differences associated with MR^5^, MMR, and MR^1^, respectively. Differences in estimated mean FACT-Leu total and TOI-FACT-Leu scores were small for MR^5^ and MMR and trivial for MR^1^. The effect size of the difference in FACT-Leu total score corresponding to MR^5^ versus MR^1^ was 0.24, which can be interpreted as small. For FACT-G total and functional well-being scores, differences associated with MR^5^ were small, and those associated with MMR and MR^1^ were trivial. MR had the weakest relationships with physical well-being and social well-being, where all differences were considered trivial.

## Discussion

Treatment with TKIs that target Bcr-Abl1 has transformed CP CML to a chronic condition with normal life expectancy in most affected patients [1]. Given the need for lifelong therapy, HRQoL from the patient perspective has become a key component of clinical decision-making [25], in addition to response to individual therapies. Although evaluation of HRQoL through PRO assessments in clinical trials for CML is limited [7], studies of bosutinib have routinely collected PRO data to assess the symptom burden and functional health status of patients [8–11]. In the phase 3 BFORE trial of bosutinib versus imatinib in patients with newly diagnosed CP CML, similar degrees of improvement or preservation of HRQoL were seen in both treatment arms after 12 months [11]. The robust PRO dataset from the BFORE trial provided an exceptional opportunity to longitudinally analyze HRQoL data in patients with CML and explore the relationship with efficacy outcomes independent of the individual TKI administered.

In the present analysis, the impact of MR with first-line bosutinib or imatinib on different dimensions of HRQoL was variable. For patients who achieved deep MR, emotional well-being, and leukemia-specific scores showed the greatest improvement, although the respective MIDs were not reached. In the initial PRO analysis from the BFORE trial, emotional well-being and leukemia-specific scores improved significantly from baseline to the 12-month time point in both the bosutinib and imatinib arms [11]. Significant improvements in emotional well-being and leukemia-specific scores were also seen in imatinib-resistant and imatinib-intolerant patients at various time points during treatment with second-line bosutinib, although correlations with efficacy in these populations have not been investigated [8, 10, 26]. Since the emotional well-being domain of the FACT-Leu questionnaire reflects patients’ optimism about their condition, a positive effect on this score with a response to treatment seems logical.

Physical and social well-being had the weakest relationships with MR in our analysis. As nearly 40% of patients with CP CML are asymptomatic at diagnosis in developed countries [27], the minimal impact of response on physical well-being was not unexpected. In addition, the physical well-being domain of FACT-Leu incorporates the impact of treatment side effects [18]. As is common for patients with CP CML receiving TKIs, most patients in the BFORE trial experienced treatment-emergent adverse events [2], which may partially explain a lack of improvement in physical well-being during treatment. Results from a prior meta-analysis of studies that included FACT-G data for patients with cancer suggested that social well-being, which measures support from family and friends and relationship fulfillment, may be less affected by disease or treatment outcomes than other FACT-G domains [28], as was seen here. The positive effect of a deep MR on emotional well-being and leukemia-specific scores would be anticipated to outweigh the influence of TKI side effects on overall HRQoL over the long term; this could be an area of future investigation.

The trivial or small effect size associated with MR for most FACT-Leu domains may reflect the already high scores (“ceiling effect”) for patients with CP CML at diagnosis, making HRQoL improvements during treatment difficult to detect even if a response is achieved. Accordingly, the prior analysis of HRQoL in the BFORE trial found no association between achievement of MMR at 12 months and FACT-Leu total score in the modified ITT population [11]. It is important to note that the FACT-Leu questionnaire was not designed to capture HRQoL features that are specific to patients with CML receiving TKI therapy and was not validated in this population [17]; thus, some items may not reflect the real-life experience of this patient group, resulting in decreased sensitivity to identify changes associated with treatment or response. Nevertheless, the current analysis indicates that even if small, there is evidence of a positive effect of response on HRQoL, with better levels of response corresponding to more noticeable HRQoL improvements. Moreover, we found here that patients with a deep MR experienced clinically meaningful changes in FACT-Leu total, FACT-G total, and TOI-FACT-Leu scores, based on the MID for each score.

Although patients with CP CML are expected to have relatively high HRQoL scores prior to treatment, especially those who are asymptomatic at diagnosis, studies have found that patients receiving long-term TKI treatment for CP CML experience inferior HRQoL than individuals in the general population [29, 30]. In one report [29], impaired HRQoL was reported in younger (aged < 60 years) and female patients compared with a matched control group; however, in patients aged ≥ 60 years who received long-term imatinib for CP CML, HRQoL was comparable to the general population. A later study [30] that found significantly compromised HRQoL in patients with CP CML who received imatinib, dasatinib, or nilotinib versus age- and gender-matched controls may have been limited by a smaller number of participants and a cross-sectional study design. Despite these findings, prospective PRO assessment in patients with CP CML treated with TKIs has indicated that HRQoL is maintained or improved from baseline during therapy [8–11, 31–34].

Response, including deep MR, to TKI treatment in patients with CP CML can be influenced by numerous factors, such as risk score, sex, adherence, and dose intensity [35]. Likewise, HRQoL can be affected by patient and treatment characteristics, including age, sex, comorbidities, and the side effects of the TKIs used to treat CML [29, 36–40]. It is also expected that these factors may influence the relationship between MR and FACT-Leu total score on an individual patient level. Although beyond the scope of the present analysis, this is a topic of interest for further research.

To our knowledge, the present analysis is the first rigorous exploration of relationships between efficacy outcomes and on-treatment HRQoL in patients with newly diagnosed CP CML from a large, prospective, multinational clinical trial. In a single-center study of 59 patients who received first-line nilotinib or imatinib in the phase 3 ENESTchina trial, optimal responses at 6 and 12 months per European LeukemiaNet guidelines [19] were associated with improvements in several HRQoL outcomes, measured with the Short Form 36 Health Survey (SF-36) questionnaire [41]. A longitudinal HRQoL analysis conducted as part of the GIMEMA trial of nilotinib for treatment of newly diagnosed CP CML reported improvements over time for physical functioning, role functioning, and fatigue, as assessed by the European Organization for Research and Treatment of Cancer Quality of Life Questionnaire-Core 30 (EORTC QLQ-C30); patients who reported greater physical fatigue prior to treatment were less likely to achieve an MMR, but association between response and on-treatment HRQoL was not assessed [33].

Analyses of HRQoL in clinical trials should be considered in the context of potential methodological issues. In the BFORE trial, PRO data were not collected from patients who discontinued from the trial, which is a common limitation of HRQoL analyses from clinical studies [25]. As of the data cutoff date for the primary analysis of the BFORE trial, 5 (1.9%) patients in the bosutinib arm and 16 (6.0%) in the imatinib arm had discontinued treatment due to suboptimal response or treatment failure [2]; thus, HRQoL could no longer be assessed longitudinally for these non-responders. In addition, there were relatively few observations in the model with an MRLR score of − 5. As previously reported, FACT-Leu questionnaire completion rates in the BFORE trial were > 80% up to month 9 in the bosutinib arm and up to month 6 in the imatinib arm [11], and thus PRO compliance was not a methodological concern here [25]. Regarding sample size considerations [25], all available data from the BFORE trial, i.e., both treatment arms, were pooled to provide a large dataset for investigation of the general relationship between MR and HRQoL.

In conclusion, we found variable impact of clinical improvement on different dimensions of HRQoL in patients with newly diagnosed CP CML in the phase 3 BFORE trial. Our results suggest that better response to TKI treatment is generally associated with improved HRQoL. For patients who achieved deep MR, emotional well-being and leukemia-specific domains showed the greatest improvement, whereas social well-being and physical well-being domains had the weakest relationships with MR; these patterns hold for patients who achieve MMR, although with diminished effect sizes. Prospective assessment of HRQoL and linking these data to clinical outcomes, including efficacy endpoints, should be systematically explored in future trials of TKIs in patients with CML.
